# Real-Time Motion Management for a Small Target in the Right Lower Lobe With Large Tumor Motion Using Radixact Synchrony: A Case Report

**DOI:** 10.7759/cureus.85195

**Published:** 2025-06-01

**Authors:** Guang-Pei Chen, An Tai, Lindsay Puckett, Eric Paulson

**Affiliations:** 1 Radiation Oncology, Medical College of Wisconsin, Milwaukee, USA

**Keywords:** lung oligometastases, markless, motion tracking, radixact synchrony, stereotactic body radiotherapy (sbrt)

## Abstract

Accurate real-time motion tracking is crucial for effective stereotactic body radiotherapy (SBRT) in lung cancer patients, particularly those with significant tumor motion. Techniques to reduce tumor motion, such as abdominal compression, breath hold, or respiratory gating, are often uncomfortable or result in longer treatment times. The Radixact Synchrony system (Accuray Inc., Sunnyvale, California, United States) enables continuous adaptation to tumor motion without the need for implanted fiducials. We report the case of a patient with a small target in the right lower lobe (RLL) with large tumor motion managed using Radixact Synchrony, highlighting treatment planning, delivery accuracy, and clinical outcomes.

## Introduction

Stereotactic body radiotherapy (SBRT) is a standard-of-care approach for inoperable early-stage non-small cell lung cancer (NSCLC) [1,2] and oligometastatic lung lesions [3]. Patients are typically planned with a four-dimensional computed tomography (4DCT) scan to assess the extent of tumor motion. This motion is usually incorporated as an additional margin to ensure that the tumor is treated during its full range of motion, forming what is known as an internal target volume (ITV). For tumors with significant motion, methods such as abdominal compression [4], breath hold [5], and respiratory gating [6] are used to minimize the volume of uninvolved lung that will receive the dose. However, abdominal compression often causes patient discomfort, and both breath hold and respiratory gating can result in longer treatment times. The CyberKnife and Radixact Synchrony systems (Accuray Inc., Sunnyvale, California, United States) use a different approach to motion management, which integrates real-time tracking with adaptive beam delivery, enabling efficient and precise dose targeting while reducing exposure to uninvolved lung tissue [7-10].

Motion management for lung cancer patients with very small lesions but with large respiratory motion may be challenging due to factors such as (1) high motion-to-size ratio, where when the lesion moves significantly (>10-15 mm) relative to its size, it becomes challenging to differentiate tumor motion from background noise in motion management system, and (2) poor tumor visibility on fluoroscopy or 2D X-ray images, where small lesions often have low lesion-to-background contrast against lung tissue, making it difficult to use fluoroscopic tracking.

This report presents the challenges and solutions associated with tracking a small target in the right lower lobe (RLL) with large tumor motion using Radixact Synchrony.

## Case presentation

A 73-year-old woman with a history of metastatic rectal cancer with oligoprogressive disease spread to the liver and lung, who previously received SBRT treatment to a right lung lesion one and a half years ago, was planned for SBRT to a new RLL lesion. The patient, with a respiratory period of approximately 6.2 seconds, underwent 4DCT simulation in head-first supine position on a Siemens Drive CT scanner (Erlangen, Germany). The 10 individual phase images were reconstructed with a slice thickness of 2 mm. The 6 mm RLL lesion demonstrated motion of 18 mm, 5 mm, and 3 mm in the superior-inferior (SI), anterior-posterior (AP), and left-right (LR) directions, respectively, based on the 4DCT. Due to prior radiation treatment and the large magnitude of tumor motion, tumor tracking with Radixact Synchrony was utilized to enable conformal dose planning and delivery.

Treatment planning

**Figure 1:**
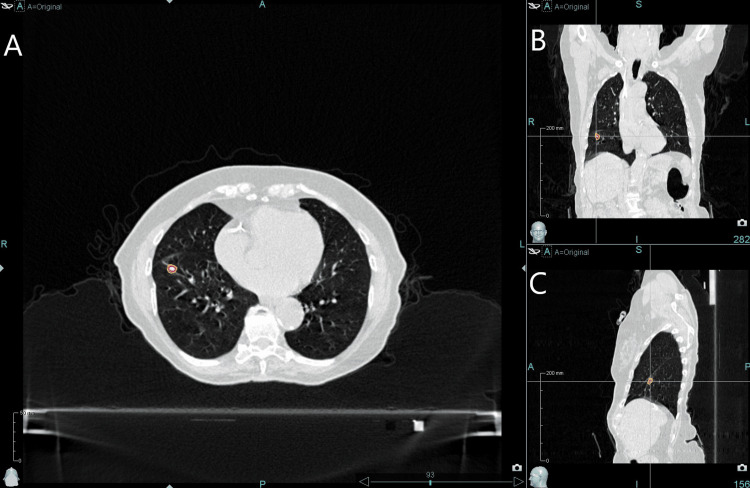
Patient's planning CT image with TTV and GTV indicated by red and orange contours, respectively, in axial (A), coronal (B), and sagittal (C) views. The image window width and level were set to the lung preset CT: computed tomography; TTV: tracking target volume; GTV: gross tumor volume

The gross tumor volume (GTV), approximately measuring 12 mm, 8 mm, and 8 mm in the SI, AP, and LR directions, respectively, was contoured based on one phase of the 4DCT, yielding a volume of 0.70 cc. The 30% phase image set was selected for treatment planning, as it represented mid-ventilation and provided optimal GTV contrast. The tracking target volume (TTV) was contoured on the 30% phase image, with window width and level adjusted from lung to soft tissue settings to improve the reliability of tracking target detection on radiographic images during treatment. The small TTV (~0.11 cc) with a low CT number (50±60 HU), large motion amplitude, and the presence of multiple adjacent lung nodules (Figure 1) posed a challenge for Synchrony tracking. The planning target volume (PTV) was generated with a 5 mm expansion of the GTV, resulting in a total volume of 5.46 cc and an SI length of 2.4 cm.

A total dose of 54 Gy was prescribed to be delivered in three fractions, following the Radiation Therapy Oncology Group (RTOG) 0236 protocol [11], ensuring robust coverage of the PTV while minimizing dose to the surrounding lung tissue.

The patient was offset laterally toward the left by approximately 3 cm, with the isocenter placed closer to the target so that the target remained within ~8 cm of the isocenter. Markerless tracking (lung with respiratory) was selected as the tracking mode. Four radiographic image angles per rotation (18°, 156°, 219°, and 326°) were chosen after careful consideration of target visibility and angle separation. The 2.5 cm dynamic jaw was used, taking into account the PTV SI length and tumor motion magnitude. A small pitch of 0.09 was set to achieve an appropriate gantry rotation period of 26.7 seconds. After optimization, a delivery with a net beam-on time of 10.41 minutes over 19.3 gantry rotations was obtained. The final modulation factor was 1.1. Adequate PTV coverage was achieved: 96.4% of the PTV received 54 Gy, with a dose heterogeneity of 66.5%. The dose distribution was conformal, with a conformality index of 1.09 and a 50% conformality index (R50) of 6.65. The maximum dose 2 cm away from the PTV was 38.2% of the prescription dose. Healthy lung tissue was spared, with V5, V20, and mean dose at 9.4%, 1.6%, and 2 Gy, respectively. The plan met all the dose constraints of the RTOG 0236 protocol for organs at risk. More detailed dose parameters, reported based on the International Commission on Radiation Units and Measurements (ICRU) 91 standard [12], are also included in Table 1.

**Table 1:** Dose parameters reported based on the ICRU 91 PTV: planning target volume; GTV: gross tumor volume; ICRU: International Commission on Radiation Units and Measurements

Contour	Dosimetric parameter	Achieved (Gy, unless otherwise specified)
PTV	D100%	48.41
	D98%	53.48
	D95%	54.54
	D50%	63.44
	D2%	82.97
GTV	D50%	78.26
	D0.035cc	84.91
Lungs	V5	9.40%
	V20	1.60%
	D0.035cc	84.83
	Dmean	2.00
Spinal cord	D0.035cc	3.77
	Dmean	0.42
Esophagus	D0.035cc	5.87
	Dmean	1.16
Heart	D0.035cc	11.93
	Dmean	2.65
Aorta	D0.035cc	5.02
	Dmean	0.61
Trachea	D0.035cc	0.18
	Dmean	0.07
Chest wall	D0.035cc	26.51
	Dmean	4.49
Rib	D0.035cc	26.37
	Dmean	11.38
Stomach	D0.035cc	0.21
	Dmean	0.10
Colon	D0.035cc	0.11
	Dmean	0.08
Liver	D0.035cc	1.13
	Dmean	0.13
Skin	D0.035cc	11.63
	Dmean	0.32

Treatment simulation

To ultimately determine whether the patient could be treated with Radixact Synchrony, a simulation plan was saved and administered to the patient four days prior to the first fraction of treatment. The simulation plan was, in principle, no different from the clinical plan, except that radiation was not delivered during simulation. One light-emitting diode (LED) marker was placed on the couch, and three LED markers were placed on the patient's abdomen in a location with visibly large motion to sample the patient's external breathing motion. A foam wedge was placed between the patient and the three LED markers, tilting the LEDs to point toward a ceiling-mounted camera throughout the couch motion range during treatment. The LED motion was modeled to relate to the internal tumor motion as determined from radiographic images. After patient setup verification and position adjustment with kV image guidance, the radiographic imaging protocol (Thorax Large, 1.6 mAs) and selected angles were entered into the system, along with modeling parameters provided by the physics team. These included the following: Potential Diff: 6 mm, Measured Delta: 5 mm, Target Offset: 30 mm, Tracking Range: 30 mm, Sensitivity: Low (setting a target detection confidence threshold of 0.45 out of 1), Target Outside Jaw Range: 10%, and Auto Pause Delay: 60 seconds. It's worth mentioning that the model building confidence, ranging from 0 to 1, has a Synchrony internal threshold of 0.25. The initial model building was fast (~30 seconds), and the model was continuously updated with newly acquired radiographic images during "beam on". Acquired radiographic images were initially assessed to confirm accurate target detection, and successive radiographic images were monitored for tracking consistency and modeling performance. The simulation processed smoothly, and the entire procedure, from patient entry to patient exit from the treatment room, was well managed within a 30-minute time slot. The patient was determined to be a good Synchrony candidate.

Treatment delivery

Treatment with tracking was conducted in the same manner as the feasibility simulation, but with actual radiation delivery. A physicist was present during both the simulation and treatment fractions to provide necessary assistance with LED setup, model building, and tracking accuracy monitoring. The patient was successfully treated on Monday, Wednesday, and Friday in the week following the simulation.

Accuracy of motion management

**Figure 2:**
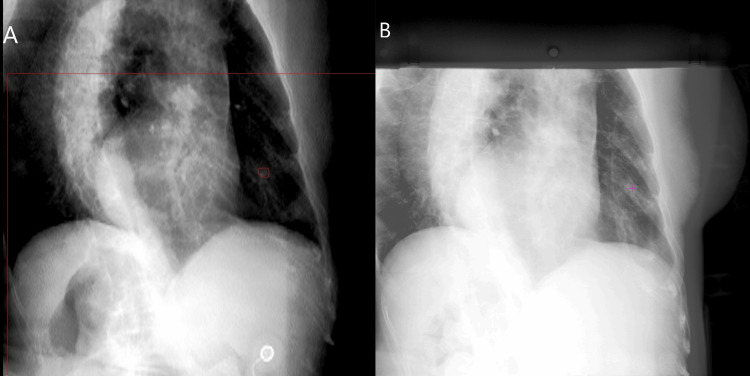
A pair of images showing motion trace: the DRR at 219° with the TTV indicated in purple (A) and a radiographic image from treatment at the same imaging angle with the detected target indicated with a purple cross (B) DRR: digitally reconstructed radiograph; TTV: tracking target volume

Radixact Synchrony continuously updated the beam position in response to real-time tumor motion, adjusting for large SI displacement and minimizing setup errors. For all three fractions of treatment, there was no treatment interruption that resulted from model-building failure. Raw data from all three fractions of the treatment were analyzed. The tracking, indicated by the target detection confidence, model building confidence, model quality (potential difference), error between model prediction and measurement (measured ∆), consistency of the tumor motion between tracking and 4DCT estimation, etc., as listed in Table 2, was found to be accurate. A digitally reconstructed radiograph (DRR) image at an image angle of 219°, along with one of its corresponding treatment radiographic images, is shown in Figure 2.

**Table 2:** Average motion management parameters from all three fractions LED: light-emitting diode

Parameter	Value
LED amplitude (mm)	8.0±2.1
Percentage of images acquired during beam on (%)	88.6
Target detection confidence	0.72±0.07
Model building confidence	0.92±0.17
Potential diff (mm)	3.6±1.9
Measured ∆ (mm)	1.5±1.2
Target motion in x (mm)	3.7±2.4
Target motion in y (mm)	13.2±4.6
Target motion in z (mm)	3.9±1.8

Clinical outcome

**Figure 3:**
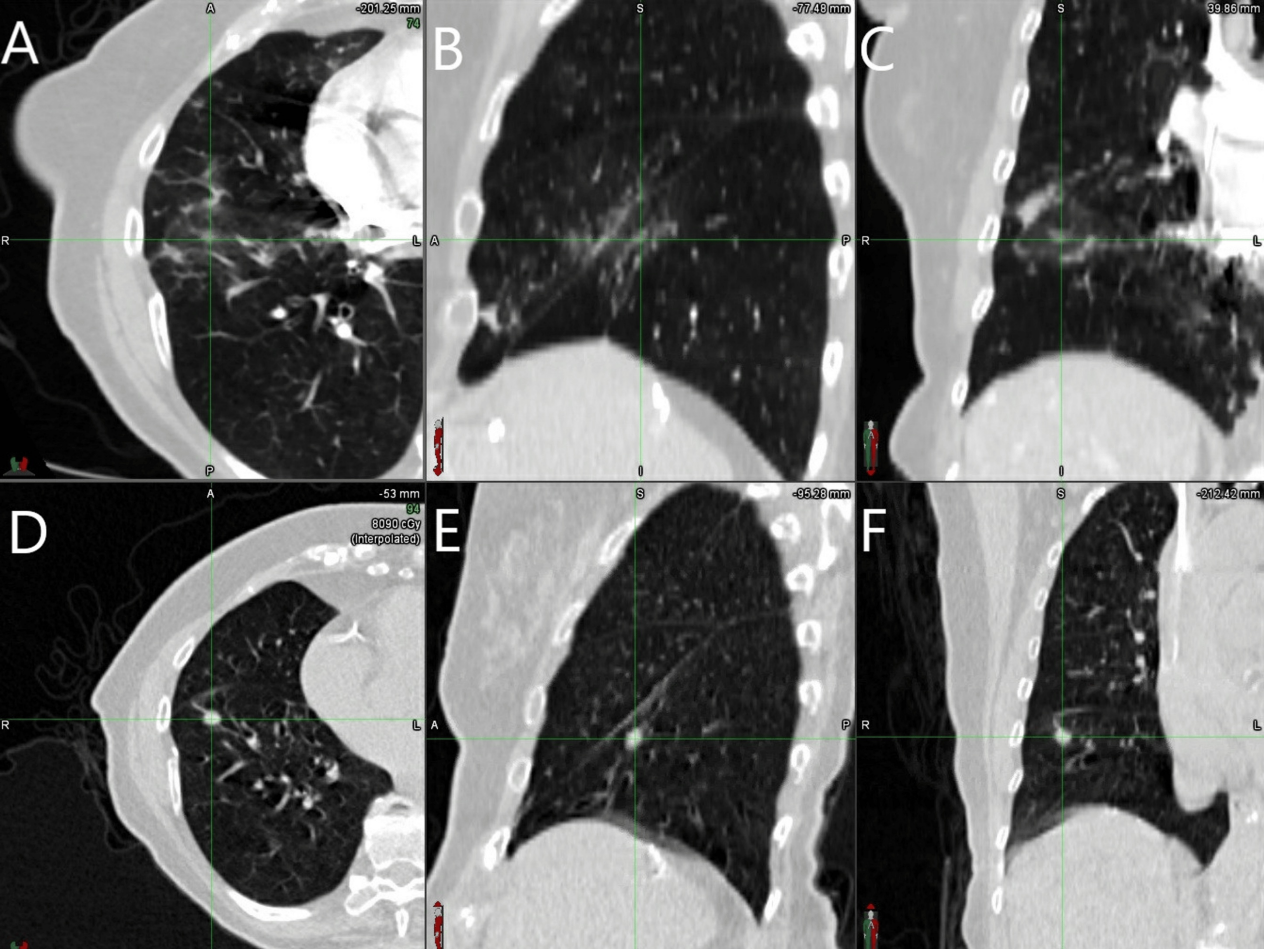
Post-treatment CT images at 20 months (A, B, C) registered with planning CT images (D, E, F), with green crosses indicating the original tumor location on axial (A and D), sagittal (B and E), and coronal (C and F) views CT: computed tomography

At the one-month follow-up after SBRT treatment with Synchrony, the patient reported feeling well. She denied any symptoms except for rare, intermittent abdominal pain (pain scale 3 out of 10), which may or may not have been related to her recent hernia surgery. She also denied any cough, shortness of breath, or other pulmonary symptoms. Post-treatment imaging at 20 months demonstrated a complete response, with no evidence of tumor progression (Figure 3). No significant changes in pulmonary nodules or nodular densities, nor significant adenopathy within the chest, have been observed since treatment.

## Discussion

Here, we detailed the planning, simulation, and SBRT treatment of a small RLL lesion with large motion using real-time target tracking with Radixact Synchrony. Implanted fiducials (e.g., gold markers) may be used to enhance visibility for small lesions; however, this invasive procedure increases patient pain and cost and carries the risk of pneumothorax. Furthermore, possible fiducial migration can compromise the accuracy of radiation treatment [13]. Careful selection of the image phase used for planning can provide optimal CT number inside the tumor for better target visibility.

CyberKnife Synchrony and Radixact Synchrony both use a similar core concept: creating a correlation model between internal target position and external respiratory surrogates to leverage real-time motion tracking for managing intrafractional tumor movement, particularly due to respiration. However, significant differences in system architecture, imaging modalities, and workflow may have led to meaningful improvements in tracking capabilities with the Radixact platform, especially for small targets without implanted fiducials. Radixact Synchrony uses a kV imaging system integrated with the treatment beam, providing the capability to image the target at any axial angle, with 2-6 angles per rotation. These angles can be adjusted based on patient anatomy and target location. In contrast, CyberKnife Synchrony relies on fixed stereoscopic kV X-ray sources at oblique angles, which can limit soft tissue contrast and hinder the visualization of small targets due to anatomical blockage, among other factors. Additionally, volumetric imaging for setup and verification on Radixact enables better initial alignment, including roll correction, and aids model building in tracking. CyberKnife does not utilize volumetric imaging for setup in the same manner. Instead, it relies on 2D X-ray image pairs and correlation with external markers or fiducials. In response to target motion, Radixact Synchrony swings the jaws in the SI direction and shifts the multileaf collimator (MLC) leaves axially. CyberKnife Synchrony, on the other hand, involves adjustments of the robotic arm but does not necessarily shift the MLC to follow the target. Recently, deep learning has been studied and shows potential to improve X-ray-based fiducial-free small lung tumor tracking on the CyberKnife Synchrony system [14].

The case described in this report highlights the efficacy of Radixact Synchrony in managing a small target with large tumor motion, an otherwise challenging scenario for conventional motion mitigation techniques. The key advantages include the following: (1) continuous real-time motion tracking without fiducials, (2) high tracking accuracy despite large motion amplitude, (3) minimized treatment margins, reducing normal lung exposure, and (4) efficient dose delivery with no significant increase in treatment time.

## Conclusions

Small lung lesions with large motion are more prone to being missed during treatment, and treatment with a large margin increases radiation exposure to healthy tissue. This case study demonstrates the clinical feasibility and effectiveness of Radixact Synchrony in managing significant respiratory-induced tumor motion for a small lesion in the lung. The system provided precise real-time motion tracking and adaptive beam delivery, ensuring accurate dose delivery despite the challenges posed by large tumor motion under low tumor visibility. The integration of motion management with image-guided adaptive radiotherapy enabled improved target conformity and minimized exposure to surrounding healthy tissue. These results highlight the value of real-time motion tracking technologies in treating mobile thoracic lesions and underscore their potential to enhance treatment precision and outcomes in complex clinical scenarios. Future studies should explore long-term outcomes and dosimetric refinements for further optimization.
